# An Automated Microfluidic Chip System for Detection of Piscine Nodavirus and Characterization of Its Potential Carrier in Grouper Farms

**DOI:** 10.1371/journal.pone.0042203

**Published:** 2012-08-09

**Authors:** Hsiao-Che Kuo, Ting-Yu Wang, Hao-Hsuan Hsu, Szu-Hsien Lee, Young-Mao Chen, Tieh-Jung Tsai, Ming-Chang Ou, Hsiao-Tung Ku, Gwo-Bin Lee, Tzong-Yueh Chen

**Affiliations:** 1 Laboratory of Molecular Genetics, Institute of Biotechnology, National Cheng Kung University, Tainan, Taiwan; 2 Translational Center for Marine Biotechnology, National Cheng Kung University, Tainan, Taiwan; 3 Agriculture Biotechnology Research Center, National Cheng Kung University, Tainan, Taiwan; 4 University Center for Bioscience and Biotechnology, National Cheng Kung University, Tainan, Taiwan; 5 Research Center of Ocean Environment and Technology, National Cheng Kung University, Tainan, Taiwan; 6 Institute of Nanotechnology and Microsystems Engineering, National Cheng Kung University, Tainan, Taiwan; 7 Department of Engineering Science, National Cheng Kung University, Tainan, Taiwan; 8 Research Division I, Taiwan Institute of Economic Research, Taipei, Taiwan; 9 Office for Energy Strategy Development, National Science Council, Taipei, Taiwan; 10 Department of Power Mechanical Engineering, National Tsing Hua University, Hsinchu, Taiwan; University of Houston, United States of America

## Abstract

Groupers of the *Epinephelus* spp. are an important aquaculture species of high economic value in the Asia Pacific region. They are susceptible to piscine nodavirus infection, which results in viral nervous necrosis disease. In this study, a rapid and sensitive automated microfluidic chip system was implemented for the detection of piscine nodavirus; this technology has the advantage of requiring small amounts of sample and has been developed and applied for managing grouper fish farms. Epidemiological investigations revealed an extremely high detection rate of piscine nodavirus (89% of fish samples) from 5 different locations in southern Taiwan. In addition, positive samples from the feces of fish-feeding birds indicated that the birds could be carrying the virus between fish farms. In the present study, we successfully introduced this advanced technology that combines engineering and biological approaches to aquaculture. In the future, we believe that this approach will improve fish farm management and aid in reducing the economic loss experienced by fish farmers due to widespread disease outbreaks.

## Introduction

Nervous necrosis virus (NNV), a piscine nodavirus, is neuropathogenic virus that results in viral nervous necrosis (VNN) and damage throughout the central nervous system [1]–[3]. NNV affects over 30 different fish species, including economically important marine fish such as groupers, cods, flounders, bass, puffers, and breams [4]. Within these fish species, grouper is a major farming fish species that has suffered from VNN in Taiwan since 1994 [5], and the resulting disease manifestiations are associated with high mortality rates (80–100%) in hatchery-reared larvae and juveniles [6]–[8]. Large-scale and concentrated fish farming industries currently experience major economic losses due to the spread of NNV-related diseases between individual fish farms.

Understanding the epidemiology of NNV is critical for controlling the spread of disease; however, the transmission pathway of NNV between different fish farms remains a mystery. Piscine nodavirus can be transmitted either vertically from the broodfish via the egg or sperm cells [9]–[11] or horizontally between individual fish [10]. Furthermore, the virus can persist for long periods of time in subclinically infected fish and remain infectious [12]. Although ozone has been applied to eliminate any remaining NNV on the surface of the eggs of groupers in seed-producing fish farms to produce virus-free eggs and juveniles, the NNV-related diseases remain an important burden to most fish farms.

The present study sought to apply the microfluidic chip system [13] to investigate NNV infection in grouper fish farms in a large number of samples and to characterize the potential carrier of the virus. In this study, the reverse transcription polymerase chain reaction (RT-PCR) was integrated into the microfluidic chip technology, which not only lowered the cost of virus detection but also shortened the analysis time. The chip is composed of acrylic and glass materials and can only be used for analysis of a single sample. The investigation began in 2002 with the isolation of NNV from different fish farms (Figure S1 and Table S1). An identical NNV strain was found in distant (30–40 km) grouper fish farms, even when the fish eggs were obtained from different suppliers. After these findings, we then started collecting fish samples from 5 grouper fish farms, and 1 of these farms was monitored intensively and sampled continuously for 7 months (October 2008–May 2009). In addition, a number of possible virus carriers were investigated in and around the fish farms, including brine shrimps, birds, *Daphnia* spp., *Ligia* spp., rotifers, *Palaemon* spp., and inlet seawater. A systematic study utilizing the microfluidic chip system was conducted to identify a major carrier of the virus outside of the fish farm and provided important information regarding the mode of virus infection inside of the fish farm.

## Results

### Performance of RT-PCR on the microfluidic chip

For comparison of the microfluidic chip RT-PCR with conventional RT-PCR, naturally NNV-infected groupers (true positives, VNN syndrome) were collected from 3 grouper fish farms in Cigu, Jiading, and Kunshen, Taiwan. NNV was detected by capillary electrophoresis (CE) and visualized by ethidium bromide staining with microfluidic chip RT-PCR and a slab gel, respectively. Of note, virus was detected in all of the fish with both methods (Table S5). Both of the methods that we used did not reveal VNN-positive signals in healthy groupers (true negatives). Serially diluted RNA templates (1×10^4^–0.25×10^1^ copies·µL^−1^) associated with NNV and specific markers were amplified and analyzed. The detection limit of the microfluidic chip was 3 copies·µL^−1^ (starting template) of direct lysis treatment (Figure S4A), which was markedly more sensitive than the detection by conventional PCR and gel visualization. There was a linear relationship between the magnitude of fluorescence signals and the viral copy number (Figure S3A). Measurement of the fluorescence intensity of NNV samples with concentrations ranging from 0–10^5^ plaque forming units (PFU)·mL^−1^ revealed that a viral concentration of 5×10^4^ PFU·mL^−1^ could be successfully detected by the microfluidic chip (Figure S3B). The detection limit of the end-point detection by fluorescent CE was estimated to be at least 10-fold greater that that of the slab-gel electrophoresis method (Figures S4A).

### Viral cDNA fragment separated by capillary electrophoresis (CE)

PCR products amplified from the samples could only be visualized on an agarose gel when the copy number exceeded 50 copies·µL^−1^ (starting template; Figure S4A). All amplicons were cloned and sequenced to confirm that they originated from NNV. The directly lysed RNA extraction method was not as effective as the use of pure RNA for the microfluidic chip template (Figure S4B), but still produced enough RNA for NNV detection. Figure S5 displays an electropherogram of the DNA markers (*Hae*III-digested ΦX174 DNA markers) and resulting RT-PCR products. Eleven DNA marker peaks and a single peak of RT-PCR nodavirus product (203 bps) were successfully separated within 2 min (Figure S5B).

### Prevalence of NNV contamination among grouper fish farms

The microfluidic chip method (Tables S5 and S6 and Figures S3, S4, and S5) was field-tested in an epidemiological investigation of 120 fish samples from 5 grouper aquaculture farms in southern Taiwan (see Tables S2, S3, and S4 and Figure S2 for the locations). NNV infection was detected in 89% (Table S6) of the samples surveyed. Four grouper fish samples from Linyuan (Tables S3, S4, Batch I) that were confirmed to be infected with NNV did not display any signs of VNN, which indicated a false positive status or presence of latent NNV infection. However, 3 grouper fish farms in Linyuan that were identified as being NNV infection-free did show signs of VNN (Table S4, Batch II and Batch III), which indicated a false negative status. The 3 false negatives and 10 true negatives produced a negative predictive value (NPV) of 77%. One hundred and three true positives and 4 false negatives resulted in a positive predictive value (PPV) of 96% (Table S6). All of the positive results obtained from microfluidic chip detection were also confirmed by plaque assay.

### NNV remained in the fish tank and contaminated newly arrived fish

We continuously monitored the same fish tank in the Linyuan indoor grouper farm (Figure S6) for 7 months (Table S4). There were 3 VNN outbreaks in December 2008 (Batch I), February 2009 (Batch II), and April 2009 (Batch III); the time between batches was approximately 3–4 weeks. The results of Batch II (NNV was detected on the second day of culturing) and Batch III (NNV was detected on the first day of culturing) demonstrated that NNV may still remain in the fish tank even after a 1-month fallow.

### Possible carrier of NNV

Due to the high detection rate of NNV measured in this study, it was of utmost importance to determine its carrier between fish farms. Therefore, we tested the possible vectors that might carry NNV between farms. Grouper fish samples (Table 1) were collected from 3 different locations (Cigu, Jiading, and Kunshen) in southern Taiwan and the possible virus carrier around/within grouper fish farms, including brine shrimps, birds (feces), *Daphnia* spp., *Ligia* spp., rotifers, *Palaemon* spp., and inlet seawater (Table 2) were tested with the microfluidic chip system. Table 1 shows that all 3 fish farms were contaminated with NNV. These 3 grouper fish farms utilized either outdoor or semi-outdoor culturing protocols (Figure S6). The results (Table 2) revealed that in 1 out of 5 and 3 out of 5 fish farms in Kunshen and Cigu, respectively, the bird feces were positive for the presence of NNV.

**Table 1:** Nervous necrosis virus (NNV) detection results from infected grouper fish from 3 different fish farms.

Location^a^	RT-PCR^b^		Symptoms^c^		After 2 weeks^d^	
	+	−	+	−	+	−
Cigu	3	0	3	0	3	0
Jiading	3	0	3	0	3	0
Kunshen	3	0	3	0	3	0

a Locations of the grouper fish farms are shown in Figure S2 and the fish samples were collected in 2008 and 2009.

b +, NNV-positive samples by microfluidic chip analysis; −, NNV-negative samples by microfluidic chip analysis.

c The first observation of viral nervous necrosis (VNN) clinical signs following sampling; +, groupers displayed VNN clinical signs; −, groupers did not display any clinical signs of disease; Clinical signs of VNN in larval-stage groupers were abnormal schooling and swimming behavior (whirling, spiraling) and loss of appetite.

d The tracking observation (disease outbreak) 2 weeks after the first observation from the same grouper fish farms; +, groupers displayed VNN clinical signs; −, groupers did not display any clinical signs of disease.

**Table 2:** Detection of potential nervous necrosis virus (NNV) carriers.

	Samples							
Location	Bird feces^a^	Rotifers	Brine shrimps^b^	*Daphnia* spp.^c^	*Palaemon* spp.	Infected grouper fish	Inlet sea water	*Ligia* spp.
	(+/−)^d^	(+/−)	(+/−)	(+/−)	(+/−)	(+/−)	(+/−)	(+/−)
Cigu	(3/2)	(0/5)	(0/5)	(0/5)	(0/5)	(5/0)	(0/5)	(0/5)
Jiading	(0/3)	(0/3)	(0/3)	(0/3)	(0/3)	(3/0)	(0/3)	(0/3)
Kunshen	(1/4)	(0/5)	(0/5)	(0/5)	(0/5)	(5/0)	(0/5)	(0/5)

a Birds commonly seen in grouper fish farms are *Egretta garzetta* (little egret), *Nycticorax nycticorax* (black-crowned night heron), and *Passer montanus* (tree sparrow).

b Brine shrimps used as a feed for larvae.

c Common water fleas.

d (number of positive/number of negative); +, NNV positive; −, NNV negative.

## Discussion

It is likely that due to the abnormality of fish farming, i.e., fish reared in greater numbers and closer proximity than those that live in the wild, viruses are more easily transferred between fish in farm environments. An example of this is found in the Japanese flounder, *Paralichthys olivaceus*, in which horizontal transmission of viral hemorrhagic septicemia virus was shown to occur between wild and farmed fish, as well as between infected and uninfected fish [14]. We have shown that the targets of the NNV preferentially replicate not only in the nervous system but also in eye and fin tissues [11]. The results from our previous work raised the possibility that the NNV infection pathway may pass through other organisms exposed to the outside environment. Importantly, before infected fish spread NNV to others, no direct evidence can typically be found regarding how the virus was transmitted to the fish farm initially. The limitation of the sensitivity of previously used detection methods may be the reason for this challenge.

In this study, we applied a recently developed microfluidic chip detection system [15], which exhibited a high analytical sensitivity (3 copies·µL^−1^ of directly lysed sample; Figure S3A) and allowed us to detect virus in small samples. Our epidemiological investigation of 120 samples from 5 grouper aquaculture farms in southern Taiwan (Table 3 and Table S2, S3, and S4) revealed the high prevalence of NNV infection (89% of the samples and 100% of the farms). Grouper aquaculture is prevalent in Southern Taiwan due to its suitable culture environment, and the cities included in our sample (Anping, Cigu, Jiading, Kunshen, and Linyuan) are the main regions of grouper farming in this region. High stocking density and high seawater temperature can also accelerate NNV disease outbreaks and result in high mortality during the rearing period [16]. In our study, hatchery-reared larvae and juvenile groupers were typically reared indoors with a stable, controlled temperature of approximately 28–29°C. Indoor rearing is a common method for intensive rearing during larval and juvenile stages in Taiwan, in which large fiberglass or concrete tanks as large as 100 m^3^ are used. Compared to outdoor rearing methods, this approach can yield a higher survival rate and permit easier handling of stock during the early stages of growth. However, the indoor approach also provides an ideal environment (i.e., stable temperature control) for NNV infection. Moreover, there is no standard fish farming procedure for treating dead fish in Taiwan; the dead fish are often dumped into drains or into the sea in the vicinity of the aquaculture farms. This practice could result in the transmission of virus in the seawater by pumping to the aquaculture farms; thus, neighboring facilities could also be at risk.

**Table 3:** Summary of the Nervous necrosis virus NNV detection results from the Linyuan^a^ grouper fish farm by integrated microfluidic chip analysis.

Experiment	RT-PCR^b^		Symptoms^c^		Number of samples
	+	−	+	−	
Batch I	30	8	28	10	38
Batch II	26	1	27	0	27
Batch III	25	2	27	0	27

a Locations of the grouper fish farms are shown in Figure S2 and the fish samples were collected in 2008 and 2009.

b +, NNV-positive samples by microfluidic chip analysis; −, NNV-negative samples by microfluidic chip analysis.

c The observation of viral nervous necrosis (VNN) clinical signs following sampling; +, groupers displayed VNN clinical signs; −, groupers did not display any clinical signs of disease; Clinical signs of VNN in larval-stage groupers were abnormal schooling and swimming behavior (whirling, spiraling) and loss of appetite.

VNN outbreaks tend to occur approximately 1 week after the first appearance of clinical signs in individual fish. However, there were examples of false positives, such as the 4 samples from Linyuan (collected on October 23, 2008, December 24, 2008, and December 25, 2008) that were identified as being NNV-infected but were actually not (Tables S3, S4). Mortality in NNV-infected grouper aquaculture farms often approaches 100% [6]–[8], and was approximately 99.7% in the present study. The few groupers that survive these outbreaks may harbor NNV and could be the source of the false positives. However, it is conceivable that the 4 aforementioned grouper fish farms might have been sampled during the NNV incubation period. In addition, 3 grouper fish samples from Linyuan were identified as NNV infection-free, but ultimately displayed symptoms of VNN syndrome (Table S4). This may have been due to sampling errors or artificial errors that occurred during transportation or experiment preparation. In the initial stages of NNV infection, some weakened fish might have already displayed symptoms of VNN while other fish remained uninfected.

The origin and mechanism of initial NNV transmission remained unclear after our preliminary analysis. Therefore, we investigated a number of possible virus carriers in and around the fish farm, including brine shrimps (feed for juveniles), birds (feces), *Daphnia* spp., *Ligia* spp., rotifers (feed for larvae), *Palaemon* spp., and inlet seawater. In our study, we could not detect the virus in inlet seawater, thus the likelihood of acquiring infection through the inlet water was negligible. This finding is in agreement with another study that could not detect any sign of NNV in inlet water [17]. Rotifers and brine shrimp do not appear to be susceptible to nodavirus (Table 3), thus decreasing the likelihood of the feed being a transmission channel. *Daphnia* spp., *Ligia* spp., and *Palaemon* spp. were also tested and showed no indication of carrying NNV (Table 2).

With regard to the other suspects were the wild birds that inhabit areas around fish farms, which have been ignored thus far as possible NNV carriers. Our data suggests that birds are potential carriers of NNV and transfer the virus between fish farms (Table 2). *Egretta garzetta* (little egret) and *Nycticorax nycticorax* (black-crowned night heron) are the most common residents around fish farms, and their food sources consist of fish, batrachians, and insects. The NNV-infected groupers are caught easily for these birds and NNV could survive in the bird's digestive system. It has been shown that nodavirus was very stable under extreme environmental conditions [18]. Once the virus has entered a fish farm or rearing unit, it may be very difficult to exterminate. Therefore, a possible transmission route may be via fish-feeding birds.

Interestingly, observations from the Linyuan grouper farm, which operated under a high security protocol including indoor farming with controlled temperatures and seawater pH and fish eggs treated with ozone, there should not be any NNV contamination in theory. However, this farm was kept virus-free for only 1 month, and was examined for NNV infection for the remaining 6 months. Consequently, we have begun further experiments to identify an alternative transmission pathway for NNV.

In conclusion, the recently developed microfluidic chip system can provide a convenient platform for large-scale NNV detection in grouper fish farms. NNV infection represents a major challenge in grouper farming. Although we only focused on 5 grouper farms in this study, the 100% NNV detection rate in 5 major grouper culturing areas revealed the serious nature of this problem. The high sensitivity of virus (3 copies) detection by this method makes the identification of the virus carrier possible, and the results point to fish-feeding birds as the main suspect of NNV transmission. However, the results from the fish farm that operated under a high security protocol indicated that there may be alternative carriers or transmission pathways involved.

## Materials and Methods

### Fish cell line and virus

The grouper cell line, GF-1 (Bioresources Collection and Research Center, Taiwan; BCRC 960094) was used for culturing and maintaining NNV. GF-1 was derived from the fin tissue of orange-spotted grouper (*E. coioides*) [19]. The cells were incubated at 28°C in antibiotic-free L15 medium (Life Technologies, Bethesda, MD, USA) supplemented with 5% v/v heat-inactivated fetal bovine serum (FBS) [20]. NNV was obtained from naturally infected grouper (*E. lanceolatus*) juveniles [21]. The virus isolates from diseased fish [2] were cultured in GF-1 cells for 5 days (until they showed a cytopathic effect [CPE]), and the 5-day cultures were submitted for NNV isolation [2].

### Virus isolation and purification

Grouper NNV (gNNV) was isolated from naturally infected groupers (*Epinephelus lanceolatus*) collected from Jiading, Taiwan in 2004 [19]. The virus was isolated from fin tissue; the tissues were frozen in liquid nitrogen and homogenated in 10 volumes of L15 medium, centrifuged at 10,000 rpm for 20 min, and the supernatant fraction was passed through a 0.22-µm filter and stored at −80°C until analysis. For collection of viral particles, the isolated virus was cultured in GF-1 cells, and the cells were collected when 90% of the cells displayed a CPE. L15 medium containing GF-1 cells and NNV was mixed with 2.2% NaCl and 5% w/v polyethylene glycol (PEG) 8,000 and centrifuged at 10,000×g at 4°C for 1 h. The pellet was resuspended in 2 mL *N*-tris(hydroxymethyl) methyl-2-aminoethanesulfonic acid (TES) buffer, mixed with an equal amount of Freon 113 and shaken vigorously for 5 min. Supernatants were combined and mixed with 3 mL, 3 mL, and 2 mL of 40%, 30%, and 20% CsCl, respectively. CsCl density gradients were formed by centrifugation in a Beckman SW40Ti rotor (Beckman Coulter, Fullerton, CA) at 35,000 rpm at 4°C for 16 h. Syringes were used to collect 3 mL of the virus-containing fraction, which was diluted 10-fold with TES buffer.

### Plaque assay

The plaque morphological assay performed on GF-1 cell monolayers was modified from Kamei et al. (1987) [22]. Serial 10-fold dilutions (10^−3^, 10^−4^, 10^−5^, 10^−6^, 10^−7^, and 10^−8^) of virus were made using L15 (supplemented with 5% FBS) medium and monolayers of GF-1 cells were inoculated in 6-well plates. Two hundred microliters of the virus dilution was added to each well and incubated at 28°C for 1 h. The virus-containing media was removed sequentially from the wells and replaced with 2 mL of diluted agarose medium (0.5% agarose solution in L15 [with 5% FBS]) and incubated at 28°C for 3 to 4 days. The cells were fixed with 2 mL fixing solution (methanol: acetic acid = 3∶1 [v/v]) at room temperature for 30 min then stained with 1% crystal violet at room temperature for 30 min. Cells were washed with phosphate buffered saline (PBS). Plates were monitored daily until the number of plaques that were counted did not change for 2 consecutive days. Plaque forming units (PFU) were calculated as follows:
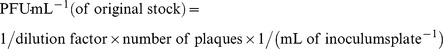



### Direct lysis, nucleic acid extraction, and RT-PCR microfluidic chip analysis

**Figure 1:**
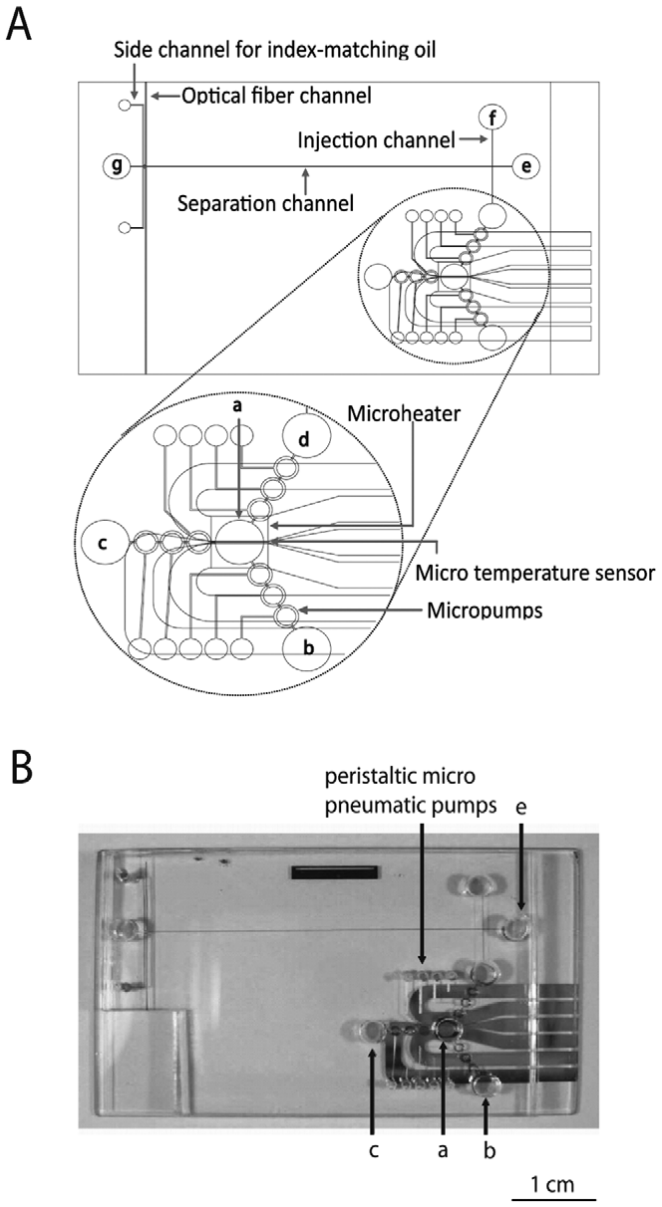
Design of integrated microfluidic chip. A. Schematic diagram of the integrated microfluidic chip. The micro RT-PCR module, capillary electrophoresis (CE) module and buried optical fibers were integrated; (a), reaction chamber; (b), RT reagent reservoir; (c), PCR reagent reservoir; (d), sample reservoir; (e), CE sample reservoir; (f), buffer reservoir; (g), waste collection chamber. B. Photograph of the microfluidic chip. The 3 layers of the integrated chip are composed of a polymethylmethacrylate (PMMA) CE chip, a glass micro PCR chip, and Polydimethylsiloxane (PDMS) micropumps. The dimension of chip is 6.5×3.9×0.8 cm^3^. The CE channel is 100 µm in width ×30 µm in depth. The volumes of the PCR and reagent chambers are 11.25 µL.

Before loading the samples onto the chip, the sample (100 mg of homogenized fish) was added to 1 mL of lysis buffer (62.5 mM Tris pH 8.3, 95 mM KCl, 3.8 mM MgCl_2_, 12.5 mM dithiothreitol, 0.63% NP-40) and centrifuged (12,000×g at 4°C for 15 min). One microliter of extracted RNA (or the supernatant of tissue lysate) was pre-heated at 70°C for 10 min in chamber A (Figure 1) with 7.5 µL reaction mixture (4.5 µL of DEPC treated water, 2 µL of Moloney Murine Leukemia Virus (M-MLV) RT 5×reaction buffer [Promega, Madison, WI, USA] and 0.5 µL of 20 µM of each primer [203-F and 203-R; Blossom, Taipei, Taiwan]). The chamber was cooled to 42°C, and 1.5 µL of the RT reaction mixture (1 µL of 2.5 mM dNTP and 0.5 µL of M-MLV Reverse Transcriptase; 200 U·µL^−1^; Promega) was automatically transferred from chamber B (Figure 1). Chamber A was maintained at 42°C for 30 min for cDNA synthesis followed by adjustment to 94°C for 2 min for enzyme deactivation. After the RT procedure, 6.5 µL of the reaction mixture containing synthesized cDNA was left in chamber a for the subsequent PCR reaction. Another micropump automatically transferred 3.5 µL of the PCR reaction mixture (1 µL of 2.5 mM dNTP, 1 µL of 10×PCR buffer with 15 mM Mg^2+^ [Violet, Taipei, Taiwan], 0.5 µL of 20 µM of each primer (203-F and 203-R), and 0.5 µL of Taq DNA polymerase; 1000 U; 5 U·mL^−1^ [Violet]) from chamber C (Figure 1) to chamber A. For PCR, chamber A was heated up to 94°C for 1 min (pre-denaturation), and then to 94°C for 10 s, 60°C for 20 s and 72°C for 20 s for 20 thermal cycles. The final cycle was followed by an additional 72°C for 1 min of post-elongation. Finally, the PCR product was automatically transferred to chamber D by the last micropump set. Other samples such as the brine shrimps, bird feces, *Daphnia* spp., *Ligia* spp., rotifers, *Palaemon* spp., and inlet seawater were analyzed by the method described above.

### Design of microfluidic chip

The integrated microfluidic chip was designed and fabricated as described in our previous work [13], [15], [17]. The scheme is shown in Figure 1. Briefly, the 6.5×4.5 cm^2^ chip consists of a micro RT-PCR module, a CE module, and 2 buried optical fibers. The micro RT-PCR module is comprised of microheaters and resistors, a microtemperature sensor, and 3 micropumps and chambers. Therefore, it can perform rapid heating (20±0.2°C·s^−1^) and cooling (10±0.2°C·s^−1^) [13], [15]. The 100-µm wide and 30-µm deep CE module is comprised of 2 polymethylmethacrylate (PMMA) structures. The lower structure houses the injection, separation, and optical fiber channels. The upper structure contains pre-drilled holes [13], [15]. The total volumes of the PCR and reagent chambers are 11.25 µL.

### Capillary electrophoresis (CE) on the microfluidic chip

Laser-induced fluorescence technology was used for detection of separated DNA molecules on the CE module of the chip [13], [15]. DNA was labeled with florescent dye using laser excitation and emission wavelengths of 491 nm and 509 nm, respectively. The RT-PCR product was kept in the chamber with same volumes of CE buffer as those used for the RT-PCR product (1.75% w/w hydroxypropylmethylcellulose [Sigma-Aldrich, St. Louis, MO, USA] in TBE with 1% v/v YO-PRO-1 fluorescent dye [Molecular Probes, Eugene, OR, USA; 24]) and 7 µL *Hae*III-digested ΦX174 DNA markers (10 ng·µL^−1^; General Electric (GE) Healthcare, Buckinghamshire, UK) were pumped from chamber F and mixed in chamber E (Figure 1). The mixture in chamber E was driven by electrokinetic forces and injected into the separation channel, where the DNA molecules were simultaneously separated and detected by the buried optical fibers [13], [15].

### Fish samples and VNN observation

Six to ten juvenile groupers (*Epinephelus coioides* and *E. lanceolatus*, reared for 25–90 days) were randomly collected from each of 5 different grouper aquaculture farms (Anping, Cigu, Jiading, Kunshen, and Linyuan; Figure S2, Table 3, and Table S2, S3, and S4) in southern Taiwan between 2008 and 2009. The samples were pooled together and homogenized in liquid nitrogen for RNA extraction.

The behavior of the grouper fish in the farms (Cigu, Jiading, Kunshen, and Linyuan; Table S3) were recorded by observing the VNN clinical signs for 2 weeks. The fish showing clinical signs were collected and further examined by RT-PCR and microfluidic chip RT-PCR (Figure 1). The clinical signs for identifying VNN were abnormal schooling and swimming behavior (whirling and spiraling), abnormal pigmentation, and loss of appetite.

### RNA isolation, cDNA synthesis, and conventional and microfluidic chip RT-PCR

RNA extraction from the homogenated whole fresh fish larvae and pure NNV particles was performed using TRIzol reagent (Molecular Research Center, Cincinnati, OH, USA) according to the manufacturer's instructions. Briefly, for RNA extraction from 100 mg tissue, 1 mL TRIzol regent was added and followed by addition of 200 µL ice-cold chloroform. RNA was precipitated by the addition of 500 µL isopropanol. RNA was subsequently redissolved in 100 µL diethyl pyrocarbonate (DEPC)-treated H_2_O. RNA that was not used immediately was stored at −80°C. Reverse-transcription was performed by M-MLV reverse transcriptase (Promega) according to the manufacturer's protocol. For the RT reaction, 2 µg of the extracted total RNA was used as a template and mixed with random primers (10 µM), dNTPmix (2.5 mM), RT buffer (5×), and reverse transcription transcriptase (200 U·µL^−1^) in a total volume of 25 µL. One microliter of cDNA from the RT reaction was used as a template and mixed with PCR buffer (10×, 5 µL; Bioman Scientific Co., Ltd., Taipei, Taiwan), dNTPmix (2.5 mM, 4 µL) (Bioman Scientific Co), specific primers pairs (203-F, GACGCGCTTCAAGCAACTC, and 203-R, CGAACACTCCAGCGACACA GCA) [11; (10 µM and 1 µL each)], Bio Taq DNA polymerase (5 U·µL^−1^, 1 µL; Bioman Scientific Co), and 37 µL of dH_2_O were used for PCR, which involved 94°C for 5 min and 35 cycles of 94°C for 40 sec, 55°C for 40 sec, 72°C for 40 sec, and 72°C for 5 min. RNA that was isolated from purified virus and cDNA were quantified using an Ultrospec 3300 Pro spectrophotometer (Amersham Biosciences, Piscataway, NJ, USA) and dilutions were made with sheared salmon sperm DNA (5 ng·mL^−1^) as a diluent.

Direct lysis and nucleic acid extraction for RT-PCR with a microfluidic chip (Figure 1) was used for detecting virus from fish and environmental samples.

### Viral copy number calculation

The viral copy number was identified by the molecular weight (1 viral genome = 1.5×10^6^ Da[ = 4542 (bps)×330(Da)]) of the virus. For 1 µg·µL^−1^ of viral RNA, there are 6.66×10^−13^ ( = 1000×10^−9^/1.5×10^6^) viral moles, equal to 4.0×10^11^ ( = 6.66×10^−13^×6.023×10^23^) virus copies.

## Supporting Information

Figure S1 **Putative coat protein sequences of the 4 isolated NNV strains from 3 fish farms (Table S1).** CG051202R2 and CG221002R2 were collected form Cigu, JD170103R2 was collected from Jiading, and KS230102R2 was collected from Kunshen, Taiwan. The amino acid differences are indicated as bold letters. The same virus was presented in 2 distant fish farms. Virus protein sequences for strains CG051202R2 and KS230102R2, isolated from Cigu and Kunshen (30 km apart), are the same. In the other case, virus CG221002R2 and JD170103R2 from Cigu and Jiading (40 km apart) have the identical RNA2 sequences.
(TIF)

Figure S2 **The locations of the grouper fish farms in Taiwan that were included in this study.** Taiwan is located between the tropical and subtropical regions. The grouper aquacultures are mainly gathered in southern Taiwan due to the preferred warm temperature of grouper fish. Dark circles indicate the 5 major regions of grouper fish farms in which our sampling took place. The white star indicates the location of the National Cheng Kung University. (Modified from a map available at http://mapsof.net under a Creative Commons Attribution-ShareAlike 1.0 License.)
(TIF)

Figure S3 **The linear relationship between the magnitude of fluorescence signals and the viral copy number.** A. The relationship between the fluorescence signals and the concentration of purified RNA (starting template, 12.5–100 copies·µL^−1^). B. The detection limit of products analyzed by the capillary electrophoresis (CE) module; fluorescence intensity for different virus concentrations and a threshold line with an amplitude of 1 mV.
(TIF)

Figure S4 **The detection of virus on slab-gel electropherograms by using direct lysis method.** A. Slab-gel electropherograms of RT-PCR products. Lane M: 100-bp DNA ladders (Yeastern Biotech Corp., Taiwan); Lanes 1–5 contain samples with different viral RNA concentrations of 10^4^, 10^3^, 10^2^, 50, and 25 (copies·µL^−1^), respectively. B. Comparison of pure RNA (isolated virus) and RNA obtained from the direct lysis method by conventional RT-PCR. Four fish were treated with different RNA extraction methods. The NNV-free control was isolated from a healthy adult grouper's fin tissue for RT-PCR; no NNV was detected by the NNV-specific primer pair (203F and 203R) from this sample. The white arrow indicates the size (203 bp) of the PCR product.
(TIF)

Figure S5 **Electropherograms of the RT-PCR products from purified RNA.** A. Slab-gel electropherograms for the amplified PCR products from fish tissues using the newly developed micro PCR module. (Lane M: 100-bp DNA ladders; 1: RT-PCR products from brain tissues of *E. lanceolatus*). B. Electropherograms of the RT-PCR products (203 bps) from purified RNA. The minimum concentration detected on the CE module was 12.5·copies µL^−1^. The mixture of DNA markers and RT-PCR products obtained from the infected grouper resulted in 11 DNA marker peaks and a single peak from the RT-PCR product (203 bps) that were successfully separated within 2 min.
(TIF)

Figure S6 **Fish farming protocols.** A. Indoor protocol; the temperature and the pH of the seawater are controlled. The fish tank is isolated from outside elements, including fish-feeding birds and sunlight. B. Semi-outdoor protocol; the fish tank is isolated from fish-feeding birds, but sunlight can penetrate inside. C. Outdoor protocol; the culturing tank is in a natural environment.
(TIF)

Table S1 **Virus isolates from infected grouper fish in four different fish farms.**
(DOC)

Table S2 **Examination of the Anping grouper fish farm for nervous necrosis virus (NNV) infection by microfluidic chip analysis.**
(DOC)

Table S3 **Examination of grouper fish farms in Cigu, Jiading, Kunshen and Linyuan for nervous necrosis virus (NNV) infection by microfluidic chip analysis.**
(DOC)

Table S4 **Three examinations (Batch I, II, and III) of the Linyuan^a^ grouper fish farm for nervous necrosis virus (NNV) infection by microfluidic chip analysis.**
(DOC)

Table S5 **Comparison of microfluidic chip and conventional RT-PCR methods for nervous necrosis virus (NNV) detection.**
(DOC)

Table S6 **Specificity of the microfluidic chip method.**
(DOC)
