# Supplementary figures and images for: An Automated Microfluidic Chip System for Detection of Piscine Nodavirus and Characterization of Its Potential Carrier in Grouper Farms
Source: PLoS One. 2012 Aug 9;7(8):e42203. doi: 10.1371/journal.pone.0042203 (PMC3415436; doi:10.1371/journal.pone.0042203)

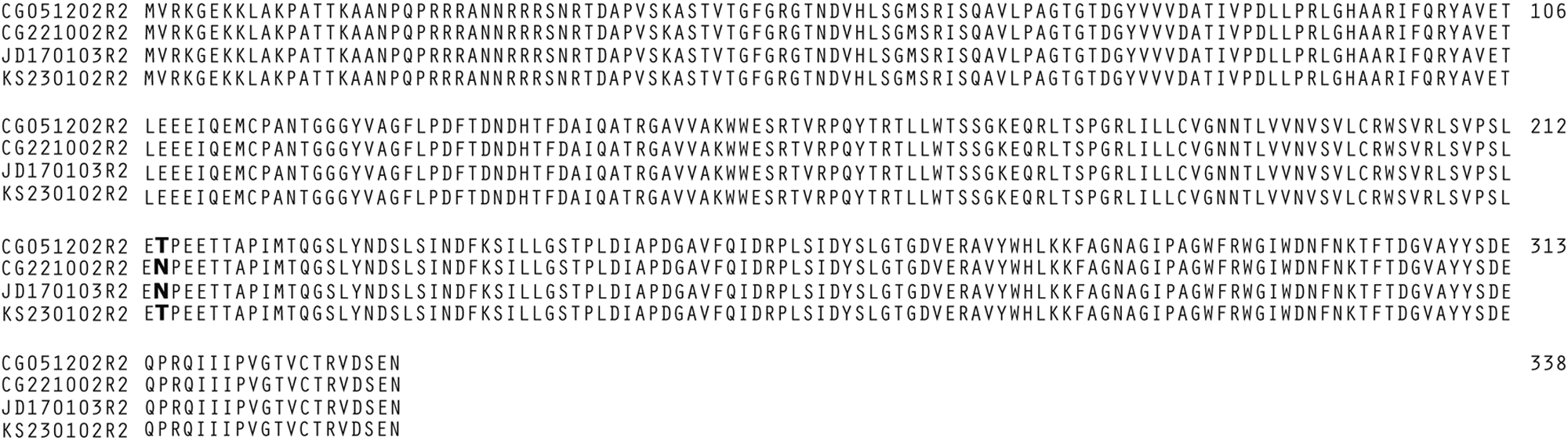

Supplement: Figure S1 — Putative coat protein sequences of the 4 isolated NNV strains from 3 fish farms (Table S1). CG051202R2 and CG221002R2 were collected form Cigu, JD170103R2 was collected from Jiading, and KS230102R2 was collected from Kunshen, Taiwan. The amino acid differences are indicated as bold letters. The same virus was presented in 2 distant fish farms. Virus protein sequences for strains CG051202R2 and KS230102R2, isolated from Cigu and Kunshen (30 km apart), are the same. In the other case, virus CG221002R2 and JD170103R2 from Cigu and Jiading (40 km apart) have the identical RNA2 sequences. (TIF) [file pone.0042203.s001.tif]

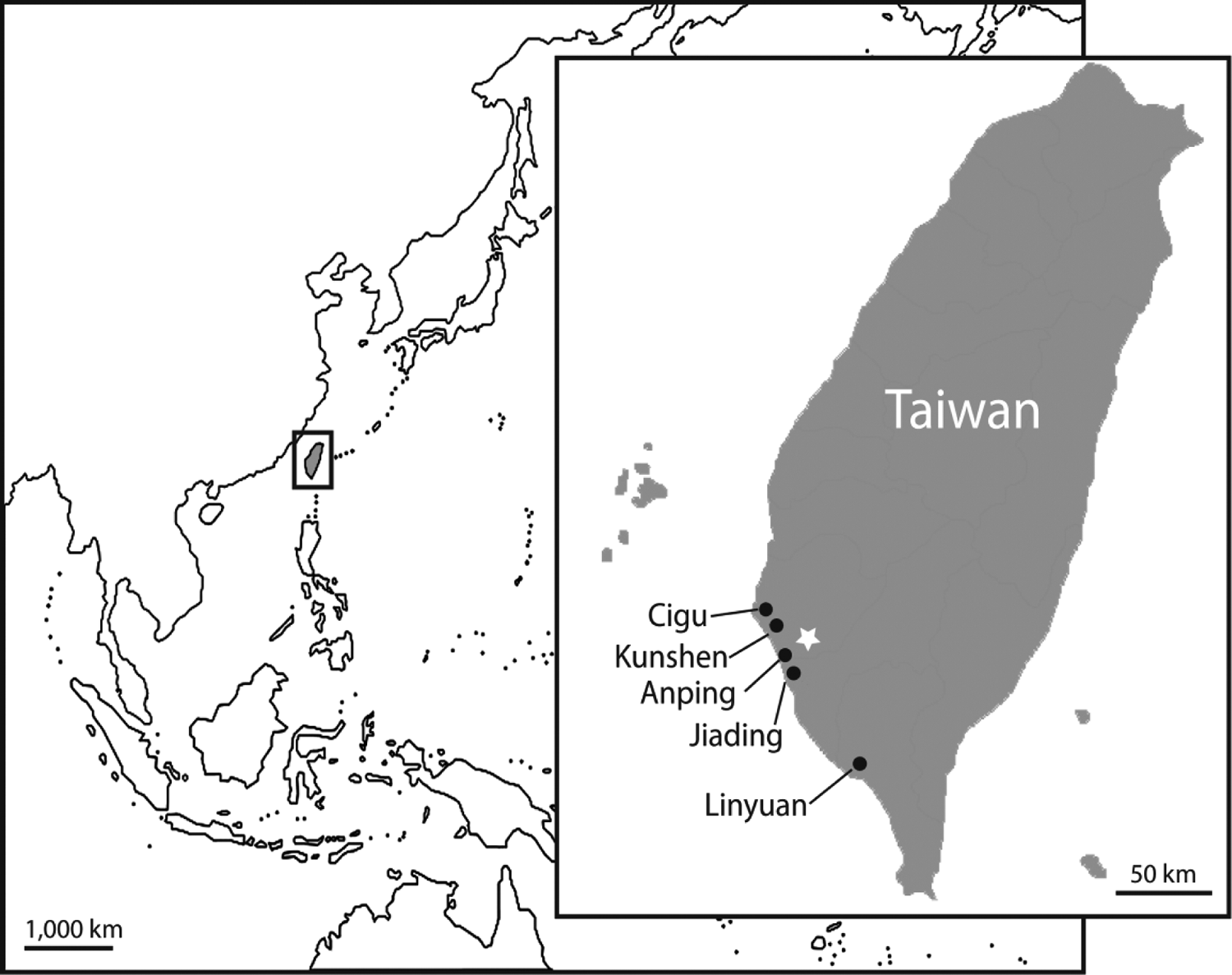

Supplement: Figure S2 — The locations of the grouper fish farms in Taiwan that were included in this study. Taiwan is located between the tropical and subtropical regions. The grouper aquacultures are mainly gathered in southern Taiwan due to the preferred warm temperature of grouper fish. Dark circles indicate the 5 major regions of grouper fish farms in which our sampling took place. The white star indicates the location of the National Cheng Kung University. (Modified from a map available at http://mapsof.net under a Creative Commons Attribution-ShareAlike 1.0 License.) (TIF) [file pone.0042203.s002.tif]

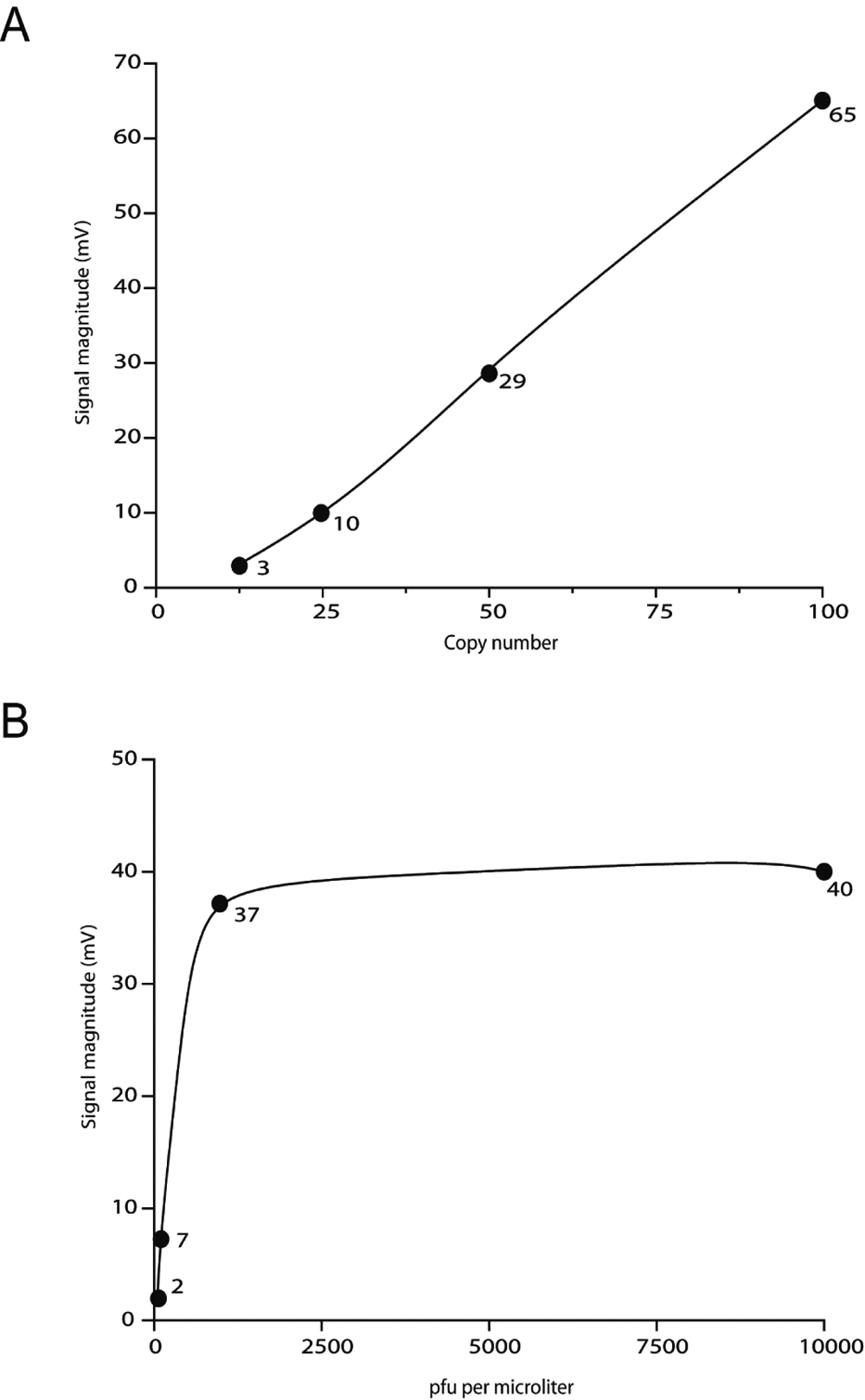

Supplement: Figure S3 — The linear relationship between the magnitude of fluorescence signals and the viral copy number. A. The relationship between the fluorescence signals and the concentration of purified RNA (starting template, 12.5–100 copies·µL−1). B. The detection limit of products analyzed by the capillary electrophoresis (CE) module; fluorescence intensity for different virus concentrations and a threshold line with an amplitude of 1 mV. (TIF) [file pone.0042203.s003.tif]

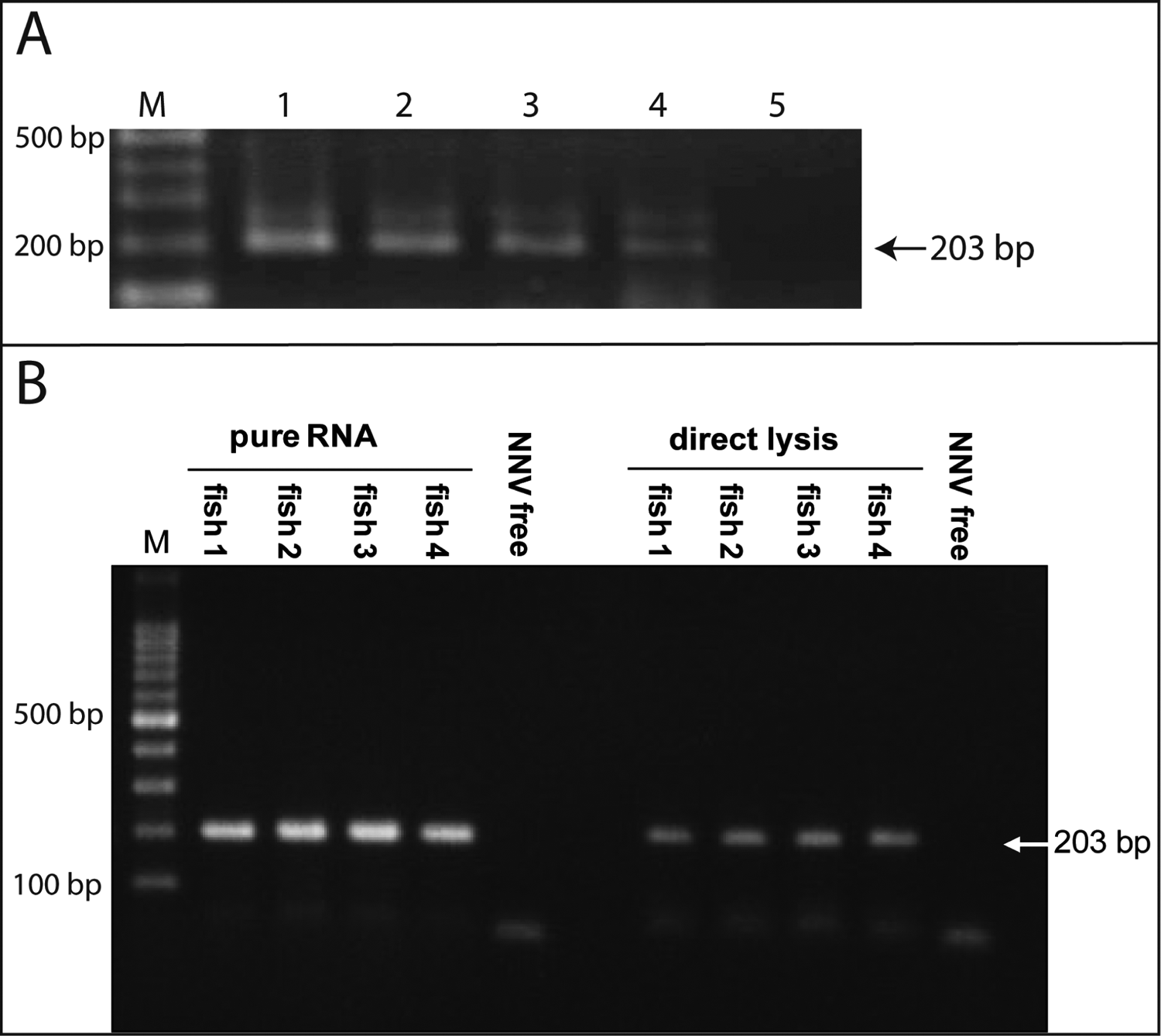

Supplement: Figure S4 — The detection of virus on slab-gel electropherograms by using direct lysis method. A. Slab-gel electropherograms of RT-PCR products. Lane M: 100-bp DNA ladders (Yeastern Biotech Corp., Taiwan); Lanes 1–5 contain samples with different viral RNA concentrations of 104, 103, 102, 50, and 25 (copies·µL−1), respectively. B. Comparison of pure RNA (isolated virus) and RNA obtained from the direct lysis method by conventional RT-PCR. Four fish were treated with different RNA extraction methods. The NNV-free control was isolated from a healthy adult grouper's fin tissue for RT-PCR; no NNV was detected by the NNV-specific primer pair (203F and 203R) from this sample. The white arrow indicates the size (203 bp) of the PCR product. (TIF) [file pone.0042203.s004.tif]

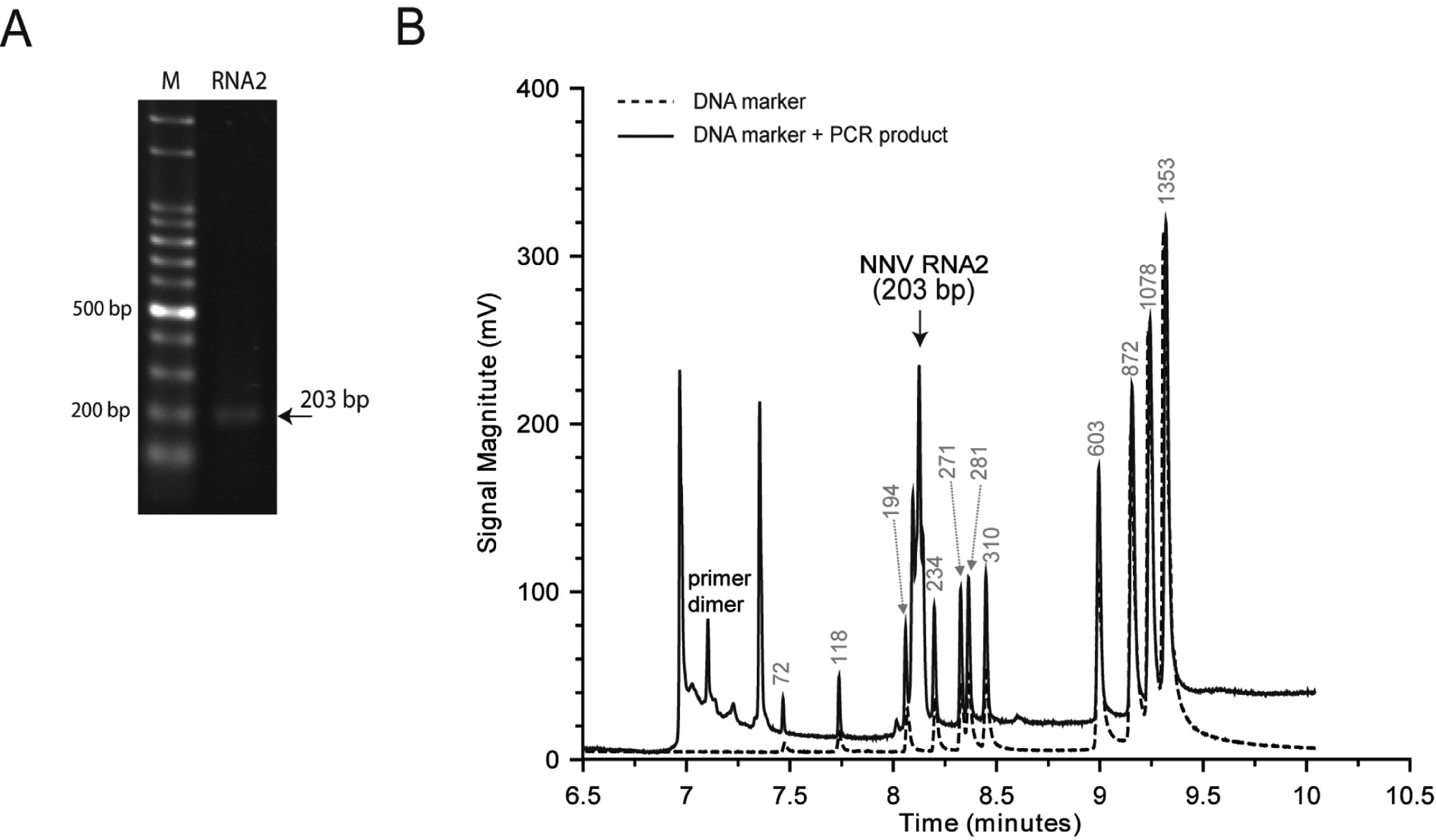

Supplement: Figure S5 — Electropherograms of the RT-PCR products from purified RNA. A. Slab-gel electropherograms for the amplified PCR products from fish tissues using the newly developed micro PCR module. (Lane M: 100-bp DNA ladders; 1: RT-PCR products from brain tissues of E. lanceolatus). B. Electropherograms of the RT-PCR products (203 bps) from purified RNA. The minimum concentration detected on the CE module was 12.5·copies µL−1. The mixture of DNA markers and RT-PCR products obtained from the infected grouper resulted in 11 DNA marker peaks and a single peak from the RT-PCR product (203 bps) that were successfully separated within 2 min. (TIF) [file pone.0042203.s005.tif]

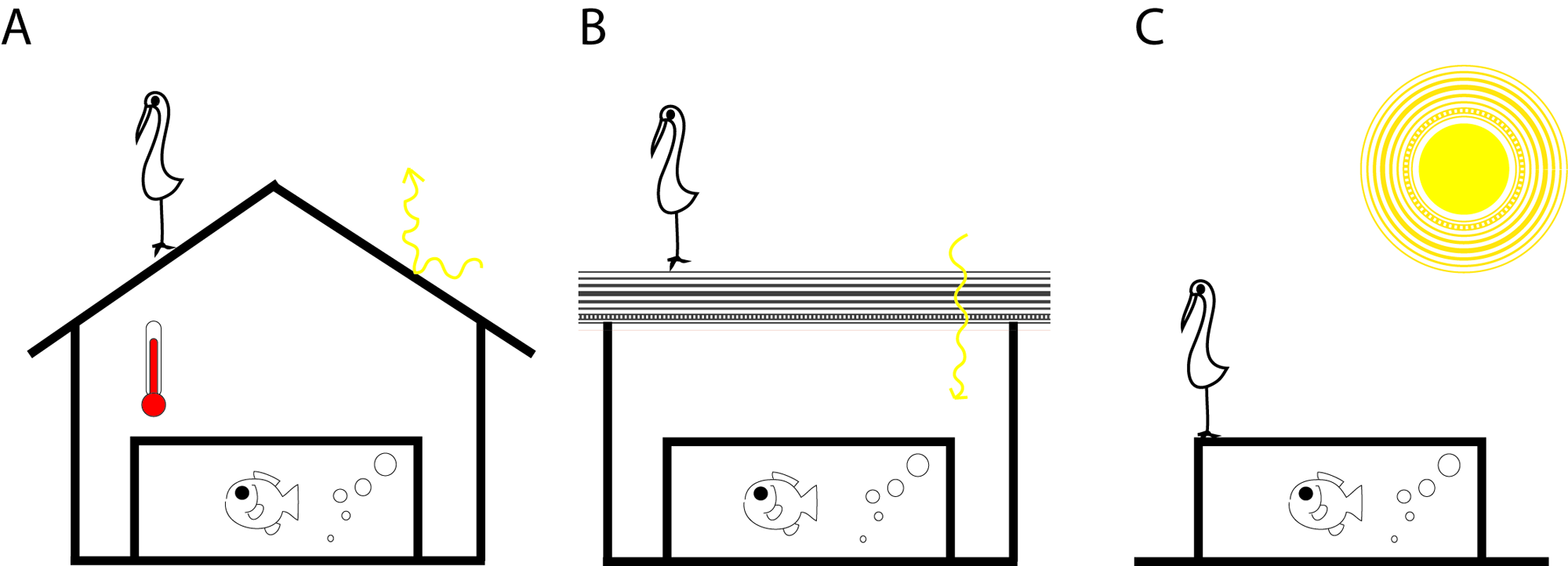

Supplement: Figure S6 — Fish farming protocols. A. Indoor protocol; the temperature and the pH of the seawater are controlled. The fish tank is isolated from outside elements, including fish-feeding birds and sunlight. B. Semi-outdoor protocol; the fish tank is isolated from fish-feeding birds, but sunlight can penetrate inside. C. Outdoor protocol; the culturing tank is in a natural environment. (TIF) [file pone.0042203.s006.tif]
