# Supplementary material for: An Automated Microfluidic Chip System for Detection of Piscine Nodavirus and Characterization of Its Potential Carrier in Grouper Farms
Source: PLoS One. 2012 Aug 9;7(8):e42203. doi: 10.1371/journal.pone.0042203 (PMC3415436; doi:10.1371/journal.pone.0042203)
Supplement: Table S1 — Virus isolates from infected grouper fish in four different fish farms. (DOC) [file pone.0042203.s007.doc]

**Table S1**. Virus isolates from infected grouper fish in 4 different fish farms.

| Isolates | Host species | Year of isolation | Location^a^ |
| --- | --- | --- | --- |
| cg221002 | Dragon grouper (*Epinephelus lanceolatus*) | 2002 | Cigu |
| cg051202 | Orange-spotted grouper (*Epinephelus coioides*) | 2002 | Cigu |
| Jd1710103 | Dragon grouper  (*Epinephelus lanceolatus*) | 2003 | Jiading |
| ks231002 | Orange-spotted grouper ( *Epinephelus coioides*) | 2002 | Kunshen |

^a^Locations of the grouper aquaculture farms are shown in Figure S2.
