# Supplementary material for: An Automated Microfluidic Chip System for Detection of Piscine Nodavirus and Characterization of Its Potential Carrier in Grouper Farms
Source: PLoS One. 2012 Aug 9;7(8):e42203. doi: 10.1371/journal.pone.0042203 (PMC3415436; doi:10.1371/journal.pone.0042203)
Supplement: Table S2 — Examination of the Anping grouper fish farm for nervous necrosis virus (NNV) infection by microfluidic chip analysis. (DOC) [file pone.0042203.s008.doc]

**Table S2. Examination of the Anping^a^ grouper fish farm for nervous necrosis virus (NNV) infection by microfluidic chip analysis.**

| RT-PCR^b^ | Date | Symptoms ^c^ |
| --- | --- | --- |
| + | 15/May/2009 | + |
| + | 20/May/2009 | + |
| + | 7/Jun/2009 | + |
| + | 19/Jun/2009 | + |
| − | 22/Jun/2009 | − |
| + | 25/Jun/2009 | + |
| + | 29/Jun/2009 | + |
| + | 1/Jul/2009 | + |
| + | 6/Jul/2009 | + |
| + | 20/Jul/2009 | + |
| + | 27/Jul/2009 | + |
| + | 27/Jul/2009 | + |
| + | 29/Jul/2009 | + |
| + | 30/Jul/2009 | + |
| + | 31/Jul/2009 | + |
| + | 1/Aug/2009 | + |

^a^Anping grouper fish farm uses the indoor protocol; The temperature was controlled at approximately 28°C and the pH of the seawater was 7.37-7.49. The grouper species was *E. lanceolatus.*

^b^Six fish (50-60 days after hatching) were collected and pooled together for microfluidic chip RT-PCR; +, indicates NNV detection; −, indicates no NNV detection.

^c^The observation of viral nervous necrosis (VNN) clinical signs following sampling; +, groupers displaying VNN clinical signs; −, groupers not displaying clinical signs; The clinical signs of VNN-infected larval-stage groupers were abnormal schooling and swimming behavior (whirling, spiraling) and loss of appetite.
