# Supplementary material for: An Automated Microfluidic Chip System for Detection of Piscine Nodavirus and Characterization of Its Potential Carrier in Grouper Farms
Source: PLoS One. 2012 Aug 9;7(8):e42203. doi: 10.1371/journal.pone.0042203 (PMC3415436; doi:10.1371/journal.pone.0042203)
Supplement: Table S3 — Examination of grouper fish farms in Cigu, Jiading, Kunshen and Linyuan for nervous necrosis virus (NNV) infection by microfluidic chip analysis. (DOC) [file pone.0042203.s009.doc]

**Table S3**. Examination of the grouper fish farms in Cigu, Jiading, Kunshen and Linyuan for nervous necrosis virus (NNV) infection by microfluidic chip analysis.

| Location | Days after hatching^a^ | Date of collection | Protocol | Temperature | RT-PCR^b^ | Symptoms ^c^ | After  2 weeks^d^ |
| --- | --- | --- | --- | --- | --- | --- | --- |
| Cigu | 50-60 | Oct 9, 2008 | outdoor | 26.5°C | + | + | + |
|  |  |  |  |  | + | + | + |
|  |  |  |  |  | + | + | + |
| Jiading | 80-90 | Aug 26, 2008 | semi-  outdoor | 27-28 °C | + | + | + |
|  |  |  |  |  | + | + | + |
|  |  |  |  |  | + | + | + |
| Kunshen | 35-40 | Aug 13, 2008 | semi-  outdoor | 28°C | + | + | + |
|  |  |  |  |  | + | + | + |
|  |  |  |  |  | + | + | + |
| Linyuan | 25-30 | Oct 23, 2008 | indoor | 29°C | + | − | − |
|  |  |  |  |  | − | − | − |
|  |  |  |  |  | + | − | − |

^a^The grouper species was *E. coioides.*

^b^Six fish were collected and pooled together for microfluidic chip RT-PCR; +, indicates NNV detection; −, indicates no NNV detection.

^c^The first observation of viral nervous necrosis (VNN) clinical signs following sampling; +, groupers displaying VNN clinical signs; −, groupers not displaying clinical signs; The clinical signs of VNN-infected larval-stage groupers were abnormal schooling and swimming behavior (whirling, spiraling) and loss of appetite.

^d^Tracking observation, 2 weeks after the first observation from the same grouper fish farms; +, groupers with VNN clinical signs; −, groupers not showing any clinical signs.
