# Supplementary material for: An Automated Microfluidic Chip System for Detection of Piscine Nodavirus and Characterization of Its Potential Carrier in Grouper Farms
Source: PLoS One. 2012 Aug 9;7(8):e42203. doi: 10.1371/journal.pone.0042203 (PMC3415436; doi:10.1371/journal.pone.0042203)
Supplement: Table S4 — Three examinations (Batch I, II, and III) of the Linyuana grouper fish farm for nervous necrosis virus (NNV) infection by microfluidic chip analysis. (DOC) [file pone.0042203.s010.doc]

**Table S4**. Three examinations (Batch I, II, and III) of the Linyuan^a^ grouper fish farm for nervous necrosis virus (NNV) infection by microfluidic chip analysis.

| RT-PCR^b^ | Collection date | Symptoms ^c^ |
| --- | --- | --- |
| Batch I | | |
| − | 16/Dec/2008 | − |
| − | 17/Dec/2008 | − |
| − | 18/Dec/2008 | − |
| − | 19/Dec/2008 | − |
| − | 20/Dec/2008 | − |
| − | 21/Dec/2008 | − |
| − | 22/Dec/2008 | − |
| − | 23/Dec/2008 | − |
| + | 24/Dec/2008 | − |
| + | 25/Dec/2008 | − |
| + | 26/Dec/2008 | + |
| + | 27/Dec/2008 | + |
| + | 28/Dec/2008 | + |
| + | 29/Dec/2008 | + |
| + | 30/Dec/2008 | + |
| + | 31/Dec/2008 | + |
| + | 1/Jan/2009 | + |
| + | 2/Jan/2009 | + |
| + | 3/Jan/2009 | + |
| + | 4/Jan/2009 | + |
| + | 5/Jan/2009 | + |
| + | 6/Jan/2009 | + |
| + | 7/Jan/2009 | + |
| + | 8/Jan/2009 | + |
| + | 9/Jan/2009 | + |
| + | 10/Jan/2009 | + |
| + | 11/Jan/2009 | + |
| + | 12/Jan/2009 | + |
| + | 13/Jan/2009 | + |
| + | 14/Jan/2009 | + |
| + | 15/Jan/2009 | + |
| + | 16/Jan/2009 | + |
| + | 17/Jan/2009 | + |
| + | 18/Jan/2009 | + |
| + | 19/Jan/2009 | + |
| + | 20/Jan/2009 | + |
| + | 21/Jan/2009 | + |
| + | 22/Jan/2009 | + |
| Batch II | | |
| − | 16/Feb/2009 | + |
| + | 17/Feb/2009 | + |
| + | 18/Feb/2009 | + |
| + | 19/Feb/2009 | + |
| + | 20/Feb/2009 | + |
| + | 21/Feb/2009 | + |
| + | 22/Feb/2009 | + |
| + | 23/Feb/2009 | + |
| + | 24/Feb/2009 | + |
| + | 25/Feb/2009 | + |
| + | 26/Feb/2009 | + |
| + | 27/Feb/2009 | + |
| + | 28/Feb/2009 | + |
| + | 1/Mar/2009 | + |
| + | 2/Mar/2009 | + |
| + | 3/Mar/2009 | + |
| + | 4/Mar/2009 | + |
| + | 5/Mar/2009 | + |
| + | 6/Mar/2009 | + |
| + | 7/Mar/2009 | + |
| + | 8/Mar/2009 | + |
| + | 9/Mar/2009 | + |
| + | 10/Mar/2009 | + |
| + | 11/Mar/2009 | + |
| + | 12/Mar/2009 | + |
| + | 13/Mar/2009 | + |
| + | 14/Mar/2009 | + |
| Batch III | | |
| + | 18/Apr/2009 | + |
| + | 19/Apr/2009 | + |
| + | 20/Apr/2009 | + |
| + | 21/Apr/2009 | + |
| + | 22/Apr/2009 | + |
| + | 23/Apr/2009 | + |
| + | 24/Apr/2009 | + |
| + | 25/Apr/2009 | + |
| + | 26/Apr/2009 | + |
| + | 27/Apr/2009 | + |
| + | 28/Apr/2009 | + |
| + | 29/Apr/2009 | + |
| + | 30/Apr/2009 | + |
| + | 1/May/2009 | + |
| + | 2/May/2009 | + |
| + | 3/May/2009 | + |
| + | 4/May/2009 | + |
| + | 5/May/2009 | + |
| − | 6/May/2009 | + |
| − | 7/May/2009 | + |
| + | 8/May/2009 | + |
| + | 9/May/2009 | + |
| + | 10/May/2009 | + |
| + | 11/May/2009 | + |
| + | 12/May/2009 | + |
| + | 13/May/2009 | + |
| + | 14/May/2009 | + |

^a^Linyuan grouper fish farm uses the indoor protocol; The temperature was controlled at approximately 29°C and the pH of the seawater was 7.37-7.49. The grouper species was *E. coioides.*

^b^Six to ten fish (25-30 days after hatching) were collected and pooled together for microfluidic chip RT-PCR; +, indicates NNV detection; −, indicates no NNV detection.

^c^The observation of viral nervous necrosis (VNN) clinical signs following sampling; +, groupers displaying VNN clinical signs; −, groupers not displaying any clinical signs; The clinical signs of VNN-infected larval-stage groupers were abnormal schooling and swimming behavior (whirling, spiraling) and loss of appetite.
