# Supplementary material for: An Automated Microfluidic Chip System for Detection of Piscine Nodavirus and Characterization of Its Potential Carrier in Grouper Farms
Source: PLoS One. 2012 Aug 9;7(8):e42203. doi: 10.1371/journal.pone.0042203 (PMC3415436; doi:10.1371/journal.pone.0042203)
Supplement: Table S5 — Comparison of microfluidic chip and conventional RT-PCR methods for nervous necrosis virus (NNV) detection. (DOC) [file pone.0042203.s011.doc]

**Table S5**. Comparison of microfluidic chip and conventional RT-PCR methods for nervous necrosis virus (NNV) detection.

| Location^a^ | Methods^b^ | |
| --- | --- | --- |
|  | mRT-PCR^c^ | RT-PCR |
| Cigu | +,+,+ | +,+,+ |
| Kunshen | +,+,+ | +,+,+ |
| Jiading | +,+,+ | +,+,+ |

^a^Locations of the grouper fish farms are shown in Figure S2. The collected (August 2008) grouper (*E. coioides*) fish already displayed clinical signs of NNV infection.

^b^Six fish were collected and pooled together for each replicate; +, indicates positive result (NNV detection) for each replicate.

^c^RT-PCR on a microfluidic chip.
