# Supplementary material for: An Automated Microfluidic Chip System for Detection of Piscine Nodavirus and Characterization of Its Potential Carrier in Grouper Farms
Source: PLoS One. 2012 Aug 9;7(8):e42203. doi: 10.1371/journal.pone.0042203 (PMC3415436; doi:10.1371/journal.pone.0042203)
Supplement: Table S6 — Specificity of the microfluidic chip method. (DOC) [file pone.0042203.s012.doc]

**Table S6**. Specificity of the microfluidic chip^a^ method.

| No. of samples detected / total no. with indicated result | | Sensitivity^d^ (%) | NPV ^b^ (%) | No. of samples detected / total no. with indicated result | | Specificity^e^  (%) | PPV ^c^ (%) |
| --- | --- | --- | --- | --- | --- | --- | --- |
| True positives (TP) | False negatives (FN) |  |  | True negatives (TN) | False positives (FP) |  |  |
| 103/106 | 3/106 | 97 | 77 | 10/14 | 4/14 | 71 | 96 |

^a^Living groupers (*E. coioides* and *E. lanceolatus* ) were collected from 5 different grouper fish farms (total of 120 fish samples; Tables S1-S6) in southern Taiwan (Anping, Cigu, Jiading, Kunshen, and Linyuan; Figure S2)

^b^NPV, negative predictive value; TN/(FN+TN)

^c^PPV, positive predictive value; TP/(TP+FP)

^d^Sensitivity = TP / (TP + FN)

^e^Specificity = TN / (FP + TN)

Abbreviations: FN, false negative; FP, false positive; NPV, negative predictive value TN/(FN+TN); PPV, positive predictive value, TP/(TP+FP); TN, true negative; FN, false negative
